# Examination of the Association Between Access to Care and Lung Cancer Screening Among High-Risk Smokers

**DOI:** 10.3389/fpubh.2021.684558

**Published:** 2021-08-25

**Authors:** Chien-Ching Li, Alicia K. Matthews, Yu-Hsiang Kao, Wei-Ting Lin, Jad Bahhur, Linda Dowling

**Affiliations:** ^1^Department of Health Systems Management, Rush University, Chicago, IL, United States; ^2^Department of Population Health Nursing Science, The University of Illinois at Chicago, Chicago, IL, United States; ^3^Department of Behavioral and Community Health Sciences, Louisiana State University Health Sciences Center, New Orleans, LA, United States; ^4^Department of Global Community Health and Behavioral Sciences, Tulane University, New Orleans, LA, United States; ^5^Department of RUMG Administration, Rush University Medical Center, Chicago, IL, United States

**Keywords:** racial disparities, lung cancer screening, low-dose computed tomography, social determinants of health, access to care

## Abstract

**Objective:** The purpose of this study was to examine the influence of access to care on the uptake of low-dose computed tomography (LDCT) lung cancer screening among a diverse sample of screening-eligible patients.

**Methods:** We utilized a cross-sectional study design. Our sample included patients evaluated for lung cancer screening at a large academic medical center (AMC) between 2015 and 2017 who met 2013 USPSTF guidelines for LDCT screening eligibility. The completion of LDCT screening (yes, no) was the primary dependent variable. The independent variable was access to care (insurance type, living within the AMC service area). We utilized binary logistic regression analyses to examine the influence of access to care on screening completion after adjusting for demographic factors (age, sex, race) and smoking history (current smoking status, smoking pack-year history).

**Results:** A total of 1,355 individuals met LDCT eligibility criteria, and of those, 29.8% (*n* = 404) completed screening. Regression analysis results showed individuals with Medicaid insurance (OR, 1.51; 95% CI, 1.03-2.22), individuals living within the AMC service area (OR, 1.71; 95% CI, 1.21–2.40), and those aged 65–74 years (OR, 1.49; 95% CI, 1.12–1.98) had higher odds of receiving LDCT lung cancer screening. Lower odds of screening were associated with having Medicare insurance (OR, 0.30; 95% CI, 0.22–0.41) and out-of-pocket (OR, 0.27; 95% CI, 0.15–0.47).

**Conclusion:** Access to care was independently associated with lowered screening rates. Study results are consistent with prior research identifying the importance of access factors on uptake of cancer early detection screening behaviors.

## Introduction

Chronic high-frequency cigarette smoking is the leading preventable cause of lung cancer worldwide (1). Lung cancer is the second most common cancer diagnosis and the leading cause of cancer-related mortality in the United States (2). In 2020, there were an estimated 228,820 new cases of lung cancer diagnosed (3). The overall 5-year survival rate for lung cancer is 18.6%, with more than half of all lung cancer patients dying within 1 year of diagnosis (4). In 2013, the National Lung Screening Trial (NLST) demonstrated low-dose computed tomography (LDCT) lung cancer screening in older smokers reduced lung cancer mortality by 15–20% due to the early detection of treatable lesions (5). Based on the results from the NLST trial, the United States Preventive Services Task Force (USPSTF) recommended annual screening with LDCT in older adults aged 55–80 years who have a 30 pack-year smoking history and currently smoke or who have quit within the past 15 years (6). In addition, the Centers for Medicare and Medicaid Services (CMS) and private insurers cover annual LDCT screening among people at high risk for lung cancer (7, 8).

Early detection of cancer through screening is an effective way of reducing cancer deaths. Healthy People 2030 sets a national objective for increasing the proportion of adults get lung cancer screened to be 7.5% (9). Despite the benefits of LDCT and increasing coverage by health care insurers, the uptake of lung cancer early detection among eligible smokers remains limited (10). The estimated percentage of qualified individuals who reported completion of LDCT screening ranged from 3.8% in the 2015 National Health Interview Survey (11) to 14.4% in the 2017 Behavioral Risk Factor Surveillance System (BRFSS) survey (12). To date, the factors contribute to the low uptake of LDCT screening among high-risk patients are not well-understood, additional research to identify the provider and patient-level barriers to engagement in screening among high-risk and eligible patients is needed (13–15). Researchers have identified provider-level barriers to patient screening, including poor clinician knowledge (e.g., lack of knowledge about screening guidelines) (16–19), concerns about screening (e.g., skepticism about evidence base and potential harms) (20–22), and time constraints prohibiting appropriate counseling and shared decision making (17, 18). Patient or individual-level barriers to lung cancer screening include fear related to lung cancer (20, 23), lack of knowledge (24), and negative attitudes and inaccurate beliefs about lung cancer screening (25). Furthermore, a range of individual-level demographics is associated with lung cancer screening. For example, older participants, single, insured, or diagnosed with cancer, were more likely to undergo LDCT screening (26). Although individual-level factors contribute to poor health-related outcomes, it has become increasingly clear factors outside of the individual are instrumental to the development and persistence of cancer health inequalities (27).

For the past decade, research to examine the influence of social determinants of health (SDOH) on a myriad of health inequalities, including cancer, has been conducted (28, 29). Social determinants of health are the environmental conditions, both social and physical, affect a wide range of risk exposures, health behaviors, and health-related outcomes (30, 31). In general, the SDOH includes five interconnected domains: economic stability, education, neighborhood, built environment, social and community context, and access to care and health care quality (18, 19). The National Institutes of Health has adopted the SDOH framework to guide research associated with health inequalities and has encouraged additional research to understand better the associations between the SDOH and health-related inequalities and the mechanistic pathways associated with these relationships (32).

Access to care represents a significant yet highly modifiable SDOH. Beyond the SDOH framework, access to health care is central to several theoretical models of health promotion. For example, Andersen's behavioral model of health services utilization has defined access to care (e.g., health insurance, proximity to healthcare facility) as one of the enabling factors related to health services utilization, including cancer screening (33). Prior research has shown poor access to health care is associated with disparities in breast, cervical, and colorectal cancer screening across various patient populations (34–36). However, limited research exists related to the influence of access to care on lung cancer screening after controlling for patient demographic characteristics (e.g., age, race, sex) and smoking behaviors (current smoking status and frequency and length of time smoked). To address this gap in the lung cancer screening literature, we examined the influence of access to care (health insurance type, proximity to healthcare facility) on the completion of LDCT lung cancer screening among patients who met the 2013 USPSTF screening eligibility guidelines. We hypothesized access to health care may be associated with LDCT lung cancer screening uptake after controlling for individual demographic and smoking variables.

## Methods

### Study Design

The study utilized a cross-sectional study design using data (2015–2017) from a prominent mid-western academic medical center (AMC). The AMC is located close to the west side of Chicago which are largely racially segregated neighborhoods of concentrated poverty and have a significant proportion of premature deaths attributed by chronic diseases and cancer (37). These neighborhoods comprise more than 500,000 individuals within the AMC's primary service area (38).

**Figure 1:**
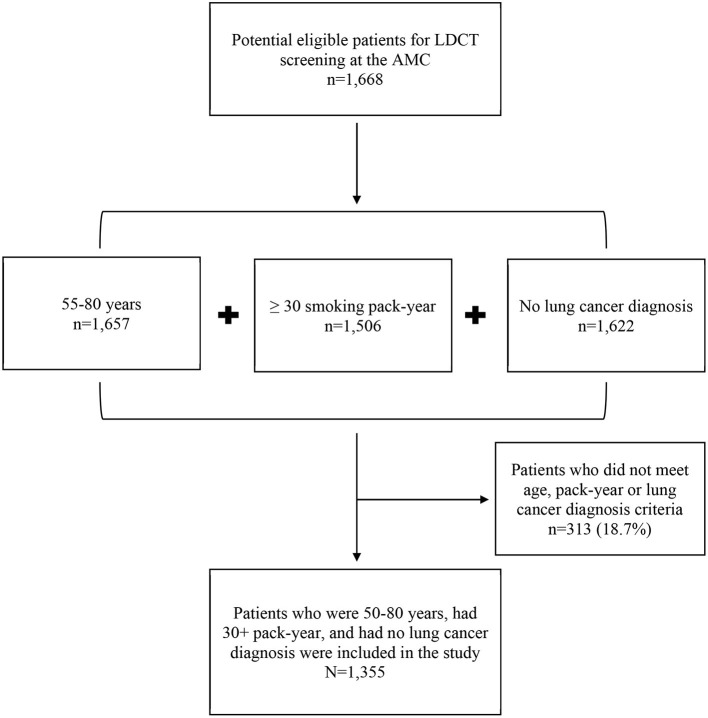
Study sample flow chart.

First, we identified potentially eligible patients for LDCT lung cancer screening at the AMC. Next, we determine which patients met the 2013 USPSTF guideline for LDCT screening (6). Eligibility criteria were: (i) aged 55–80 years, (ii) no diagnosis of lung cancer or lung-related symptoms, (iii) either a current or former smoker, and (iv) reporting a 30+ pack-year smoking history. We were unable to identity smokers who quit smoking within 15 years due to limited data collection. Therefore, we eliminated 313 patients (18.7%) who did not meet screening criteria. The final analytical dataset included *N* = 1,355 patients (see Figure 1). The Institutional Review Boards (IRB) of the Rush University Medical Center approved the study.

### Lung Cancer Screening Program

Rush University launched its lung cancer screening program in 2015. The program aimed to increase primary care physicians and other providers assess lung cancer screening eligibility among their active patients who smoke. Providers were trained to determine their patients' eligibility for LDCT screening based on USPSTF guidelines, complete shared decision-making about LDCT with the patient, and place an order for LDCT screening. In the program, two registered nurse navigators provide administrative oversight. They review patient eligibility, track results, address patient questions, and coordinate care for patients requiring additional imaging or procedures.

### Measures

#### Independent Variable

Access to health care (a critical social determinant of health) was the primary independent variable. In the current study, we measured two indicators of health care access: primary insurance type (Medicare, Medicaid, private insurance, out-of-pocket) and whether the patient lived within the AMC service area (yes, no). The AMC service area (39) included patients residing in the following residential zip codes 60607, 60608, 60612, 60622, 60623, 60624, 60639, 60644, 60647, and 60651. Proximity to healthcare settings is an established indicator of access to care (40).

#### Dependent Variable

Patient completion of LDCT lung cancer screening (yes, no) following the determination of eligibility was the primary outcome measure. We verified lung cancer screening completion via chart review.

#### Control Variables

Demographic factors and smoking behaviors which are known to be associated with cancer screening behaviors, were study control variables. Demographic factors included age (in years), race/ethnicity (African American, White, Other race/ethnicity), and sex (male or female). Smoking history included current smoking status (former smoker, current smoker) and the number of smoking pack-years. We calculated the number of smoking pack-years by multiplying the average number of cigarettes smoked per day by the number of years smoked (6).

### Statistical Analysis

Descriptive statistics, including frequency, percentage, mean, and standard deviation (S.D.), were used to describe the characteristics of the study sample. We conducted bivariate tests to examine the associations between LDCT screening completion and independent and control variables. Further, we stratified analysis by race/ethnicity to identify any different variables associated with LDCT screening completion between Whites, African Americans, and Other race/ethnicity. Finally, we conducted binary logistic regression analyses to examine the influence of access to care on LDCT screening completion in three regression models. The first model examined the influence of access to care on LDCT screening completion (model 1) without adjusting for covariates. In the second model, demographic factors were adjusted in the model to examine the association between access to care on LDCT screening completing (model 2). Lastly, demographics and smoking variables were adjusted together in the model to examine the extent to which access to care affects screening completion (model 3).

We performed all statistical analyses and data management using SAS software version 9.4 (SAS Institute, Cary, NC).

## Results

### Characteristics of Study Participants

Table 1 displays the characteristics of study participants. A total of 1,355 patients were eligible for LDCT screening between 2015 and 2017. Eligible patients were on average 66.3 years of age (SD = 6.2), male (50.4%), Caucasian (66.0%), former smokers (56.5%), and reported a mean smoking pack-year history of 51.5 years (SD = 42.3).

**Table 1:** Characteristics of the study sample (*N* = 1,355).

	**Overall (*n* = 1,355)**	**LDCT completion**		***p*-Value**
		**Yes (*n* = 404, 29.8%)**	**No (*n* = 951, 70.2%)**	
		***N* (%)**	***N* (%)**	
**DEMOGRAPHIC FACTORS**				
**Age (Years) [Mean** **±** **SD]**	66.3 ± 6.2	65.8 ± 6.2	66.5 ± 6.3	0.049^*^
**Age**				0.291
55–64	583 (43.0)	186 (31.9)	397 (68.1)	
65–74	591 (43.6)	170 (28.8)	421 (71.2)	
75–80	181 (13.4)	48 (26.5)	133 (73.5)	
**Sex**				0.593
Male	683 (50.4)	199 (29.1)	484 (70.9)	
Female	672 (49.6)	205 (30.5)	467 (69.5)	
**Race/Ethnicity**				0.005^**^
White	894 (66.0)	248 (27.7)	646 (72.3)	
African American	350 (25.8)	128 (36.6)	222 (63.4)	
Others	111 (8.2)	28 (25.2)	83 (74.8)	
**ACCESS TO HEALTHCARE FACTORS**				
**Insurance type**				<0.001^***^
Medicare	454 (33.5)	78 (17.2)	376 (82.8)	
Medicaid	142 (10.5)	70 (49.3)	72 (50.7)	
Private	645 (47.6)	241 (37.4)	404 (62.6)	
Out-of-Pocket	114 (8.4)	15 (13.2)	99 (86.8)	
**Lives within the AMC service area**			<0.001^***^	
Yes	222 (16.4)	92 (41.4)	130 (58.6)	
No	1133 (83.6)	312 (27.5)	821 (72.5)	
**SMOKING HISTORY**				
**Smoking status**				0.002^**^
Former	766 (56.5)	202 (26.4)	564 (73.6)	
Current	589 (43.5)	202 (34.3)	387 (65.7)	
**Smoking pack-years [Mean** **±** **SD]**	*51.5* ±*42.3*	*46.9* ±*21.7*	*53.4* ±*48.4*	<0.001^***^
**Smoking pack-years**				0.017^*^
30–39	417 (30.8)	139 (33.3)	278 (66.7)	
40–49	478 (35.3)	150 (31.4)	328 (68.6)	
50+	460 (33.9)	115 (25.0)	345 (75.0)	

*SD, standard deviation.*

* *p < 0.05,*

** *p < 0.01,*

*** *p < 0.001*.

### Bivariate Analyses

Less than a third of all eligible participants (29.8%, *n* = 404) completed LDCT lung cancer screening. As shown in Table 1, patients who received screening were younger (65.8 ± 6.2) than those who did receive screening (66.5 ± 6.3). African American ethnicity (36.6%), Medicaid insurance (49.3%), who lived within the AMC service area (41.4%), current smoker (34.3%), and patients who reported a 30–39 pack-year smoking history (33.3%) were correlates of lung cancer screening completion. Table 2 presents LDCT screening completion rates stratified by racial/ethnic group. Variations in correlates of screening uptake were observed based on race/ethnicity. Among African Americans, a higher percentage of patients with Medicaid (50.8%), who lived within the AMC service area (43.5%), and who were current smokers (42.6%) received screening. For Whites, patients with Medicaid (49.2%) were more likely to complete screening. None of the demographic, access to care factors, or smoking variables was associated with LDCT completion among individuals from the combined other race category. Regardless of racial/ethnic, patients who received screening had a lower smoking pack-year than those who did not receive screening.

**Table 2:** LDCT screening completion rates stratified by race/ethnicity.

	**White (** ***N*** **=** **894)**			**African American (** ***N*** **=** **350)**			**Other Race/Ethnicity (N=111)**		
	**LDCT completion**			**LDCT completion**			**LDCT completion**		
	**Yes (*n* = 248)**	**No (*n* = 646)**		**Yes (*n* = 128)**	**No (*n* = 222)**		**Yes (*n* = 28)**	**No (*n* = 83)**	
	***N* (%)**	***N* (%)**	***p*-Value**	***N* (%)**	***N* (%)**	***p*-Value**	***N* (%)**	***N* (%)**	***p*-Value**
**DEMOGRAPHIC FACTORS**									
**Age (years) [Mean** **±** **SD]**	65.9 ± 6.2	66.6 ± 6.2	0.162	65.6 ± 6.4	66.5 ± 6.5	0.195	65.8 ± 5.3	66.3 ± 6.3	0.664
**Age**			0.719			0.184			0.709
55–64	106 (28.2)	270 (71.8)		66 (41.8)	92 (58.2)		14 (28.6)	35 (71.4)	
65–74	114 (28.2)	290 (71.8)		45 (31.9)	96 (68.1)		11 (23.9)	35 (76.1)	
75–80	28 (24.6)	86 (75.4)		17 (33.3)	34 (66.7)		3 (18.8)	13 (81.3)	
**Sex**			0.550			1.000			0.247
Male	124 (26.8)	338 (73.2)		53 (36.8)	91 (63.2)		22 (28.6)	55 (71.4)	
Female	124 (28.7)	308 (71.3)		75 (36.4)	131 (63.6)		6 (17.7)	28 (82.4)	
**ACCESS TO HEALTHCARE FACTORS**									
**Insurance type**			<0.001^***^			<0.001^***^			0.053
Medicare	49 (16.2)	254 (83.8)		26 (21.1)	97 (78.9)		3 (10.7)	25 (89.3)	
Medicaid	30 (49.2)	31 (50.8)		34 (50.8)	33 (49.3)		6 (42.9)	8 (57.1)	
Private	157 (35.7)	283 (64.3)		67 (44.4)	84 (55.6)		17 (31.5)	37 (68.5)	
Out-of-Pocket	12 (13.3)	78 (86.7)		1 (11.1)	8 (88.9)		2 (13.3)	13 (86.7)	
**Living within AMC's service area**		0.060			0.032^*^			0.247	
Yes	25 (38.5)	40 (61.5)		60 (43.5)	78 (56.5)		7 (36.8)	12 (63.2)	
No	223 (26.9)	606 (73.1)		68 (32.1)	144 (67.9)		21 (22.8)	71 (77.2)	
**SMOKING HISTORY**									
**Smoking Status**			0.069			0.020^*^			0.816
Former	131 (25.3)	386 (74.7)		53 (30.5)	121 (69.5)		18 (24.0)	57 (76.0)	
Current	117 (31.0)	260 (69.0)		75 (42.6)	101 (57.4)		10 (27.8)	26 (72.2)	
**Smoking pack-years [Mean±SD]**	48.1 ± 20.9	52.1 ± 31.2	0.026^*^	45.0 ± 22.2	57.2 ± 82.6	0.039^*^	45.8 ± 26.3	53.7 ± 32.6	0.251
**Smoking pack-years**			0.127			0.119			0.311
30–39	81 (32.0)	172 (68.0)		46 (35.7)	83 (64.3)		12 (34.3)	23 (65.7)	
40–49	88 (27.8)	229 (72.2)		54 (42.9)	72 (57.1)		8 (22.9)	27 (77.1)	
50+	79 (24.4)	245 (75.6)		28 (29.5)	67 (70.5)		8 (19.5)	33 (80.5)	

*SD, standard deviation.*

* *p < 0.05,*

** *p < 0.01,*

*** *p < 0.001*.

### Multivariate Analyses

Table 3 displays the results of hierarchical logistic regression models. In Model 1, we examined the influence of access to care (insurance type and living within the AMC service area) on LDCT screening completion. Compared to patients with private insurance, Medicaid patients were more likely to complete screening (adjusted OR = 1.48; 95% CI = 1.02–2.15), while Medicare patients (adjusted OR = 0.34; 95% CI = 0.25–0.45) and out-of-pocket patients (adjusted OR = 0.26; 95% CI = 0.15–0.45) were less likely to complete screening. Patients living within the AMC service area were more likely to complete screening (adjusted OR = 1.79; 95% CI = 1.31–2.45) than those living outside the AMC service area. Model 2 shows the influence of smoking variables (current smoking status and smoking pack-year history) and access to care on LDCT screening. After adjusting for smoking status and smoking pack-year history, access to health care variables, including insurance coverage, and living within the AMC service area remained significantly associated with screening completion. In model 3, we entered demographic variables (age, race, and sex) along with smoking and access to care factors. The influence of insurance type and living within the AMC service area variables on screening completion were consistent with the results of Models 1 and 2 (Nagelkerke's R-square = 0.125; Hosmer and Lemeshow Goodness-of-Fit Test, *p* = 0.372). Among demographic variables, age was the only statistically significant predictor of screening completion. Specifically, people aged 65–74 (adjusted OR = 1.49; 95% CI = 1.12–1.98) were more likely to receive screening than those aged 55–64.

**Table 3:** Influence of access to healthcare, smoking history, and demographic factors on LDCT screening completion.

	**Model 1**		**Model 2**		**Model 3**	
	**Adjusted OR (95% C.I.)**	***P*-value**	**Adjusted OR (95% C.I.)**	***P*-value**	**Adjusted OR (95% C.I.)**	***P*-value**
**ACCESS TO HEALTHCARE FACTORS**						
**Insurance type**						
Private	Ref.		Ref.		Ref.	
Medicaid	1.48 (1.02–2.15)	0.038^*^	1.47 (1.01–2.14)	0.043^*^	1.51 (1.03–2.22)	0.033^*^
Medicare	0.34 (0.25–0.45)	<0.001^***^	0.35 (0.26–0.48)	<0.001^***^	0.30 (0.22–0.41)	<0.001^***^
Out-of-pocket	0.26 (0.15–0.45)	<0.001^***^	0.27 (0.15–0.48)	<0.001^***^	0.27 (0.15–0.47)	<0.001^***^
**Living within AMC service area**						
No	Ref.		Ref.		Ref.	
Yes	1.79 (1.31–2.45)	<0.001^***^	1.78 (1.30–2.44)	<0.001^***^	1.71 (1.21–2.40)	0.002^**^
**SMOKING HISTORY**						
**Current smoking status**						
Former			Ref.		Ref.	
Current			1.25 (0.98–1.60)	0.077	1.28 (1.00–1.64)	0.054
**Smoking pack-year**						
30–39			Ref.		Ref.	
40–49			0.98 (0.73–1.32)	0.915	0.96 (0.72–1.29)	0.802
50+			0.82 (0.60–1.11)	0.200	0.76 (0.55–1.04)	0.089
**DEMOGRAPHIC FACTORS**						
**Age**						
55–64					Ref.	
65–74					1.49 (1.12–1.98)	0.006^**^
75–80					1.48 (0.97–2.25)	0.069
**Race/Ethnicity**						
White					Ref.	
African American					1.10 (0.81–1.49)	0.548
Others					0.78 (0.49–1.26)	0.312
**Sex**						
Female					Ref.	
Male					0.97 (0.76–1.25)	0.817

* *p < 0.05,*

** *p < 0.01,*

*** *p < 0.001*.

## Discussion

The study analyzed data obtained from a large mid-west AMC serves a diverse patient population to examine the influences of access to care on completion of LDCT lung cancer screening. In particular, study results showed insurance type and proximity to healthcare were significantly associated with LDCT lung cancer screening uptake. Furthermore, access to care had a more significant impact on screening completion than individual demographics and smoking history.

In the current study, LDCT completion rates among eligible patients were low, with less than one-third of eligible patients receiving a screening test. The low uptake of screening is notable because all patients were at elevated risk of lung cancer based on their chronic and high-frequency smoking history. In addition, low screening uptake among eligible smokers is particularly concerning given a quarter of the sample was African American, a population with known lung cancer disparities. For example, in Cook County, where Chicago is, the 5-year lung cancer incidence rates among African Americans are elevated compared to whites (41), especially in communities characterized by concentrated disadvantage, racial segregation, and poor access to health care. Further, the all-cause morbidity and mortality due to smoking are higher among low-income and African American smokers due to a high prevalence of illnesses exacerbated by smoking (e.g., diabetes) (42). Thus, persistent smoking-related inequalities underscore the importance of identifying and reducing barriers to lung cancer screening among diverse patient populations.

Prior research has shown access to health care is an essential social determinant of health. In particular, proximity to a screening facility seems to influence cancer screening behaviors (43, 44). In the current study, access to health care was associated with LDCT lung cancer early detection screening after controlling other demographic and smoking variables. Specifically, patients who reported living within the AMC service area were more likely to engage in lung cancer screening than those outside these boundary areas (41.0 vs. 27.5%). These study results are consistent with previous study findings proximity to the screening center was one of the most critical factors associated with adherence to cancer early detection screenings (45–47).

Type of insurance coverage was another important indicator of healthcare access. In this study, patients reporting Medicaid insurance coverage had a higher likelihood of completing LDCT lung cancer screening than privately insured individuals. In 2014, Medicaid expansion was enacted under the Patient Protection and Affordable Care Act. Medicaid expansion provides coverage for eligible low-income individuals who do not have health care insurance (48). Medicaid expansion has improved access to care among low-income individuals (49, 50). More specifically, studies have found Medicaid expansion was associated with increased cancer screenings among low-income adults (51–54). Illinois is one of the states with early implementation of Medicaid expansion (55). According to the U.S. Census Statistics, 18.4% of Chicago city residents live at or below the poverty rate (56). Additional research is needed to understand better the role of Medicaid coverage in increasing LDCT completion rates.

In addition, we found individuals with Medicare were less like to complete LDCT screening. There are several explanations for this association. A large proportion of Medicare beneficiaries are people aged 65 years and older. This older population might have more severe comorbidities or have a short life expectancy. Therefore, healthcare provider might not recommend this group of older people to be screened, given potential risks may outweigh the benefits of screening in this population (57). Furthermore, CMS requires a mandated shared decision-making visit between the provider and Medicare beneficiary before the screening can be ordered and performed (7). Shared decision-making can improve patients' knowledge of the benefits and potential harms of LDCT screening and help in making patient-centered decision through patient-provider communication (20). However, a recent study showed only about 7% of patients who underwent LDCT screening had a shared decision-making visit (58). As such, the requirement for a separate shared decision-making visit may be a barrier (59, 60) to the uptake of LDCT screening among Medicare beneficiaries. Additional research is needed to evaluate whether the mandated shared decision-making appointment represents an unanticipated barrier to screening and identify other factors associated with potentially lower LDCT engagement among Medicare beneficiaries.

Disparities in the utilization of preventive healthcare services persist based on demographic factors. In the present study, participant age was a statistically significant correlate of screening among eligible patients. Our results were consistent with prior research showing older participants (aged 65–69) were most likely to be screened for lung cancer compared to younger participants (aged 55–59 or 55–64) (26, 61). In addition, our study found no difference in LDCT screening among people aged 75–80. One potential explanation is older adults with an anticipated life expectancy of fewer than 10 years may not be recommended for cancer screening by providers (62). Counter to prior research findings related to the influence of race/ethnicity on engagement in cancer screening (63–65), in the current study, a higher percentage of African Americans completed LDCT screening compared to white and members of other racial/ethnic groups. The AMC's lung cancer program aims to increase health screening and to improve health outcomes among people living in underserved communities (66–68). The medical center is immediately adjacent to a predominately low-income and African American community on the west side of Chicago. As an anchor institution on the West Side of Chicago, the medical center continuously works with the low-income communities to help residents address the causes of poor health and achieve better health (69, 70). These targeted initiatives may have resulted in increased interest and willingness to receive screenings among eligible patients. The observed racial differences in LDCT screening were no longer present after controlling whether patients lived within the AMC's serving areas. These findings suggest the importance of community-level outreach and engagement efforts for increasing screening behaviors among underserved communities.

## Limitations

We acknowledge several limitations within our study. First, smoking behaviors used to determine an individual's eligibility for LDCT screening were self-reported. However, all official eligibility assessments for LDCT lung cancer screening are self-reported. As such, any recall bias is likely equally distributed across all study participants. Second, the study sample size of eligible individuals for LDCT screening among former smokers might be slightly over or under-estimated due to an absence of verifiable information on how long it has been since participants quit smoking. Third, the study sample included patients seen in a large AMC located in a Midwestern state. As a result, our study results may not generalize to patients who receive services in other types of health care settings. Further, reported screening completion rates may be inaccurate due to other comorbidities (e.g., heart diseases, other cancers or severe lung diseases like asthma or chronic obstructive pulmonary disease) would exclude eligible patients from screening (71), or patients may have completed LDCT screening at another healthcare facility. Further study can examine the influence of comorbidities on screening behavior. Finally, other factors influencing cancer screening behavior such as having a usual source of care (72), access to transportation (73, 74), health literacy (75, 76), doctor's recommendation (77, 78), and other socioeconomic factors (e.g., marital status, education, income, poverty level, home rental, etc.) (79–81) were not measured due to data limitation and can be controlled in future studies.

## Conclusions

Our study highlights the influence of a critical social determinant of health, healthcare access, and lung cancer screening uptake among eligible patients. These results are consistent with prior research suggesting the relative importance of access on engagement with a range of cancer screening behaviors (34–36). Therefore, additional efforts to identify which health care coverage serves as a barrier to obtaining lung cancer screening among eligible patients are needed. Further, offering high-quality screening in different locations may reduce barriers to cancer screening.

## Data Availability Statement

The data analyzed in this study is subject to the following licenses/restrictions: Rush IRB will not allow the dataset to be shared with people outside of study team.

## Ethics Statement

The studies involving human participants were reviewed and approved by Rush University Institution of Review Board. Written informed consent for participation was not required for this study in accordance with the national legislation and the institutional requirements.

## Author Contributions

C-CL and AM: study concept and design, interpretation of study results, and manuscript write-up. Y-HK and W-TL: data analysis and interpretation of study results. JB and LD: acquisition of data and critical revision of the manuscript. All authors contributed to the article and approved the submitted version.

## Conflict of Interest

The authors declare that the research was conducted in the absence of any commercial or financial relationships that could be construed as a potential conflict of interest.

## Publisher's Note

All claims expressed in this article are solely those of the authors and do not necessarily represent those of their affiliated organizations, or those of the publisher, the editors and the reviewers. Any product that may be evaluated in this article, or claim that may be made by its manufacturer, is not guaranteed or endorsed by the publisher.
